# Optimisation of areca nut husk-derived cellulose nanofibers for enhancing the mechanical properties of epoxy composites using response surface methodology

**DOI:** 10.1038/s41598-025-11415-x

**Published:** 2025-07-19

**Authors:** Vasudevan Alagumalai, Verma Rajamanickam, Heba G. Mohamed, Irfan Anjum Badruddin, Muhammad Nasir Bashir, Rhoda Afriyie Mensah

**Affiliations:** 1 Department of Mechanical Engineering, Saveetha School of Engineering, Saveetha Institute of Medical and Technical Sciences, Chennai, 602105 India; 2 Department of Mechanical Engineering, Cambridge institute of Technology, Bangalore, Karnataka 560036 India; 3 Department of Electrical Engineering, College of Engineering, Princess Nourah bint Abdulrahman University, P.O. Box 84428, 11671 Riyadh, Saudi Arabia; 4 Mechanical Engineering Department, College of Engineering, King Khalid University, 61421 Abha, Saudi Arabia; 5 Department of Mechanical Engineering, University of Malaya, 50603 Kuala Lumpur, Malaysia; 6 Multi-Scale Fluid Dynamics Lab, Department of Mechanical Engineering, Yonsei University, 120-749 Seoul, Korea; 7 Division of Structural and Fire Engineering, Department of Civil, Environmental and Natural Resources Engineering, Luleå University of Technology, 97187 Luleå, Sweden

**Keywords:** Cellulose nanofibers, Areca nut husk, Epoxy, Composites, Nanocomposites, Response surface methodology, Mechanical properties, Agro-waste valorization, Engineering, Materials science

## Abstract

Areca nut husk, a widely available yet underutilised agro-waste, is explored in this study as a novel and sustainable source of cellulose nanofibers (CNFs), addressing both environmental concerns and the growing demand for bio-based reinforcements in polymer composites. CNFs were extracted using alkali and bleaching treatments followed by ultrasonication, yielding fibres with a mean diameter of 35.84 nm. Epoxy composites were fabricated with CNF loadings ranging from 0.1 to 1.0 wt%, while key processing parameters, including mixing time (10–30 min) and curing temperature (60–100 °C), were optimised using Response Surface Methodology (RSM) based on a Box–Behnken Design. The developed regression models exhibited high predictive accuracy, with R^2^ values of 99.63% for tensile strength and 99.80% for flexural strength. Analysis of variance (ANOVA) identified CNF content as the most influential factor (F = 934.48 and 1646.71 for tensile and flexural strength, respectively), followed by mixing time and curing temperature. Optimised conditions of approximately 1.5 wt% CNF, 22 min mixing, and 80 °C curing yielded experimentally validated tensile and flexural strengths of 61.88 MPa and 74.36 MPa, respectively, deviating by only 5.27% and 2.76% from model predictions. These results confirm the effectiveness of process-optimised CNF incorporation in enhancing mechanical performance and highlight the potential of areca nut husk as a viable, high-performance bio-reinforcement for next-generation green composites.

## Introduction

The growing interest in sustainable and high-performance materials has driven the scientific community toward exploring renewable and biodegradable alternatives to synthetic fillers in polymer composites^1,2^. Among these, cellulose nanofibers (CNFs) have emerged as a leading candidate owing to their high aspect ratio, excellent mechanical stiffness, biodegradability, and abundance in plant biomass^3,4^. CNFs can act as highly efficient reinforcements in polymer matrices by providing superior interfacial bonding and stress transfer, thereby enhancing the mechanical strength, toughness, and thermal stability of composites^5–7^. However, the mechanical performance of CNF-reinforced systems strongly depends on processing parameters, dispersion techniques, and the intrinsic characteristics of the nanofibers themselves. While substantial research has focused on CNF extraction from sources such as banana pseudostem, sugarcane bagasse, rice husk, jute, and coconut coir, limited attention has been given to areca nut husk, a lignocellulosic byproduct abundantly available in South and Southeast Asia^8,9^. Annually, millions of tons of areca nut husk are discarded or burned, leading to waste accumulation and carbon emissions^10,11^. Despite its high cellulose content (≈ 40–50%), low ash, and favourable lignin-to-cellulose ratio, the areca nut husk has seen limited application in material science, particularly in nanocellulose research^12–14^. The conversion of this agro-waste into value-added reinforcement for polymer matrices not only contributes to waste minimization and pollution control but also supports the development of circular bioeconomy strategies^15–17^. Notably, existing studies on areca nut husk-derived CNFs have been limited to basic characterisation and single-variable performance assessments, with little attention given to statistically optimising fabrication parameters for their integration into polymer matrices. In contrast, the present study not only valorises areca nut husk into CNFs using a hybrid chemical-mechanical method but also systematically investigates the impact of CNF content, dispersion time, and curing temperature on composite mechanical performance using a statistically robust Response Surface Methodology (RSM). This dual focus on underutilised biomass and data-driven process optimisation represents a novel contribution toward sustainable composite engineering. The outcomes are anticipated to support circular bioeconomy goals while enabling the development of green epoxy composites suitable for structural applications.

Recent investigations have demonstrated that nanocellulose extracted from various agro-waste sources can substantially enhance the mechanical performance of thermosetting polymer matrices. For example, in the study of Siva et al.^18^, CNFs derived from Cissus quadrangularis improved the tensile strength of PLA-based composites by approximately 13%, increasing from 58.12 MPa in microfiber-only systems to 65.68 MPa in hybrid composites containing 2 wt% nanofibers. Additionally, a marginal increase in flexural strength was observed, rising from 108.5 MPa to 110 MPa, while impact strength exhibited a more significant enhancement of 21.5%. These property improvements are primarily attributed to the nanofibers’ high aspect ratio, which allows them to occupy interfacial voids, improve stress transfer, and strengthen the fiber–matrix interface. Despite these advances, very few studies have systematically optimized the processing parameters for nanocellulose-reinforced epoxy systems using statistical modeling. Furthermore, the combined influence of CNF content, mixing time, and curing temperature has rarely been addressed in a unified framework, particularly for CNFs derived from areca nut husk. Additionally, while epoxy resins provide excellent stiffness and dimensional stability, their inherent brittleness and low fracture toughness necessitate reinforcement with nanofillers. Incorporating CNFs into epoxy matrices improves crack resistance, enhances load-bearing capacity, and contributes to ductile failure mechanisms, provided that nanofibers are uniformly dispersed and sufficiently bonded to the matrix.

In this context, the present study aims to fill a critical gap in the literature by investigating areca nut husk as a source of cellulose nanofibers for epoxy reinforcement and optimizing the process parameters to maximize mechanical performance. The research objectives are fivefold: (i) to extract CNFs from areca nut husk using alkali treatment, bleaching, and ultrasonic fibrillation; (ii) to fabricate epoxy nanocomposites with CNF loadings ranging from 0.1 to 1.0 wt%; (iii) to systematically study the effects of CNF content, mixing time (10–30 min), and curing temperature (60–100 °C) on the tensile and flexural properties of the composites; (iv) to develop predictive regression models using RSM based on a Box–Behnken Design (BBD); and (v) to experimentally validate the optimized conditions and confirm the statistical model’s accuracy. The experimental design comprises 20 runs with three independent variables, and the resulting data are analyzed using ANOVA, regression diagnostics, and surface plot analysis. The optimization is conducted through a desirability function approach, which allows simultaneous maximization of both tensile and flexural strength. This work not only validates areca nut husk as a valuable bioresource but also provides a scalable, environmentally friendly route for producing nanocomposite materials with tailored mechanical performance. Furthermore, the methodology presented here establishes a blueprint for optimizing bio-based nanocomposites through a combination of material innovation and statistical process control.

## Materials and methods

### Source of materials

For the fabrication of the epoxy-based composites, a bisphenol-A-based epoxy resin (LY556) and polyamine hardener (HY951) were used. These materials were procured from Herenba Instruments & Engineers, Chennai, India. The areca nut husk used as the source material for CNF extraction was collected from local agricultural farms in Pollachi, Tamil Nadu, India (Latitude: 10.6588° N, Longitude: 77.0085° E), a region known for its extensive areca nut cultivation. The plant material was authenticated by the Department of Agricultural Engineering, Saveetha School of Engineering, Saveetha Institute of Medical and Technical Sciences (SIMATS), Chennai, India. A voucher specimen (ID: SIMATS-AGR/ARECA/2024/014) has been deposited in the departmental repository for future reference and verification. The subsequent preparation and processing of the areca nut husk to obtain CNFs are described in detail in Sect. 2.1.

### CNF extraction from areca nut husk

Areca nut husks were first washed thoroughly with tap water to remove surface impurities and dried under ambient conditions (28–32 °C, 60–70% relative humidity) for seven days to reduce the initial moisture content. Once dried, the husks were manually chopped and ground using a high-speed domestic mixer-grinder for 10 min to obtain fibrous powder. The resulting particles had lengths ranging from 0.5 to 10 mm and an average diameter of approximately 74 ± 48 μm, as determined by optical microscopy. The first stage of chemical treatment involved alkali pulping to remove hemicellulose and part of the lignin, enhancing fibre defibrillation. Specifically, the fibrous powder was immersed in a 5% (w/v) sodium hydroxide (NaOH) solution at 80 °C for 4 h, using a solid-to-liquid ratio of 1:20 (w/v) to ensure sufficient reagent diffusion and reaction. During this process, the fibres were periodically stirred at 200 rpm to maintain suspension and uniform exposure. After alkali treatment, the fibres were washed repeatedly with deionised water until the pH of the filtrate stabilised between 6.5 and 7.5, indicating the removal of residual alkali.

**Fig. 1:**
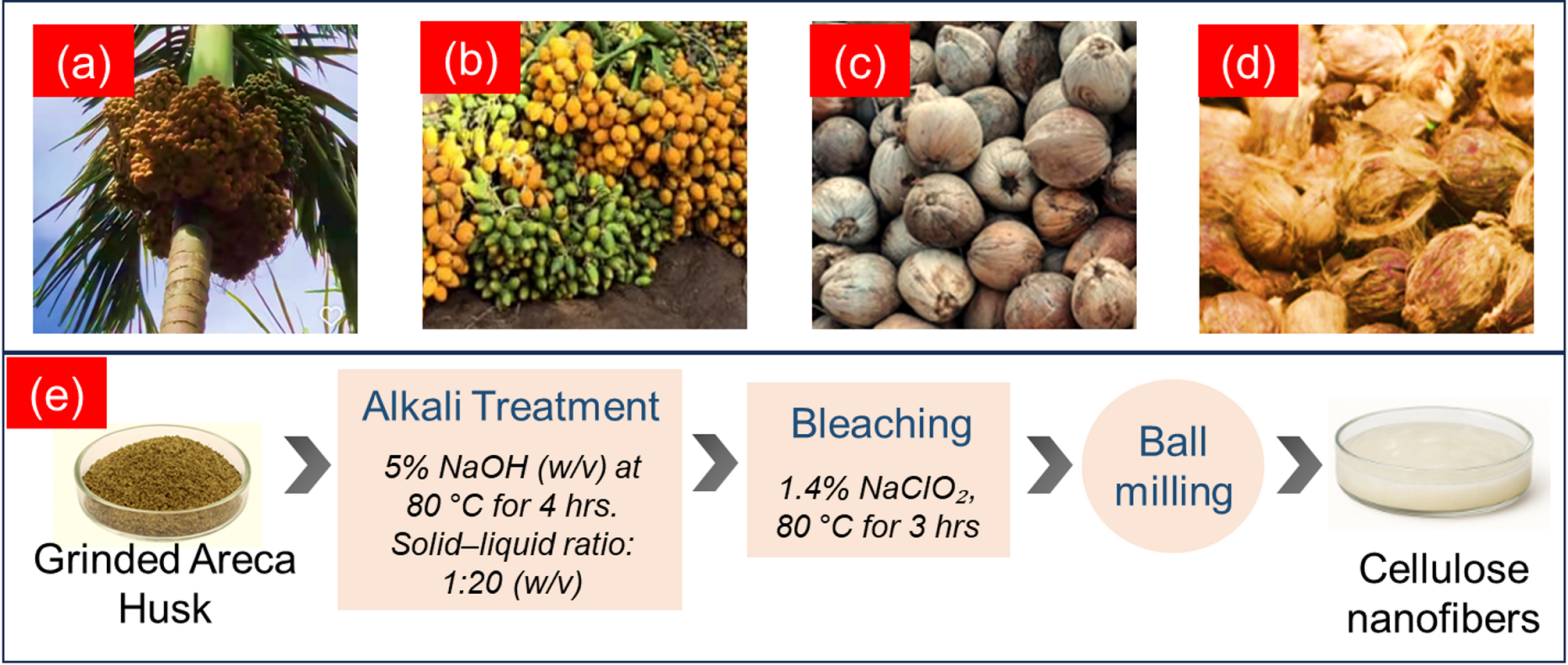
CNF preparation process.

The second stage involved bleaching for delignification, carried out using a 1.4% (w/v) sodium chlorite (NaClO₂) solution in an acetate buffer (pH ~ 4.8, prepared using acetic acid and sodium acetate) at 80 °C for 3 h. This step was repeated twice to ensure complete removal of residual lignin, which was confirmed visually when the fibres turned uniformly white. The bleached fibres were again rinsed with deionised water and oven-dried at 70 °C for 12 h until they reached a constant weight. To convert the purified cellulose into nanofibers, the dried material was re-dispersed in deionised water to form a 5% (w/v) slurry. The dispersion was then subjected to mechanical fibrillation using a laboratory-scale planetary ball miller at 750 rpm for 30 min. The mechanical shearing action broke down the macrofibrils into nanoscale fibres, resulting in a stable CNF suspension. This CNF suspension was stored in an airtight polypropylene container at 4 °C to prevent microbial growth and maintain dispersion stability until further use in composite fabrication. Figure 1 shows the process involved in the synthesis of CNF.

**Fig. 2:**
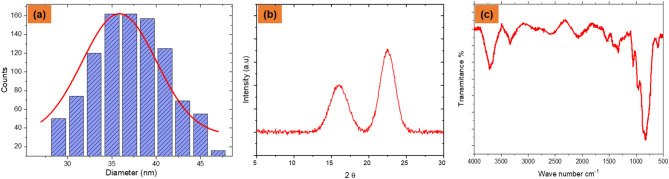
(**a**) Particle size distribution, (**b**) X-ray diffraction (XRD) pattern, (**c**) Fourier-transform infrared (FTIR) spectrum.

Figure 2 illustrates key characterisations of CNFs. The particle size distribution (Fig. 2a) follows a near-Gaussian curve, indicating uniform fibrillation critical for consistent dispersion in composites. The XRD pattern (Fig. 2b) shows a dominant peak around 22°, attributed to the (200) plane of cellulose I, confirming preserved crystallinity with minor amorphous content, essential for maintaining stiffness and structural integrity. The FTIR spectrum (Fig. 2c) reveals characteristic O–H and C–H stretching bands near 3300 cm⁻¹ and 2900 cm⁻¹, along with C–O–C vibrations near 1050–1150 cm⁻¹, indicative of purified cellulose. Absence of peaks near 1740 cm⁻¹ suggests successful removal of hemicellulose and lignin, enhancing the compatibility of CNF with polymer matrices.

### Fabrication of epoxy-CNF composites

The epoxy matrix was prepared using a commercial bisphenol-A-based epoxy resin and a hardener in a weight ratio of 10:1. The CNF suspension was introduced into the epoxy resin at varying loadings (0.1 wt% to 1.0 wt%) based on the design of experiments. A dual dispersion strategy involving magnetic stirring followed by ultrasonication was used to ensure uniform distribution of CNFs in the matrix. After complete mixing, the hardener was added, and the mixture was further degassed under vacuum to eliminate trapped air. The prepared mixture was cast into standard test moulds and subjected to curing at varying temperatures and durations as defined by the RSM design. The cured samples were post-cured at room temperature for 24 h before mechanical testing. Table 1 shows the process parameters and their coded levels for RSM design.

**Table 1:** Process parameters and their coded levels for RSM Design.

Parameter	Coded level − 1	Coded Level 0	Coded Level + 1
CNF content (wt%)	0.10	0.55	1
Mixing time (min)	10	20	30
Curing remperature (°C)	60	80	100

### Response surface methodology and analysis of variance

RSM is a widely applied statistical and mathematical approach for optimizing processes where several independent variables influence one or more response variables. It facilitates the understanding of the interactions among variables and the development of predictive models, thus aiding in the optimization of complex systems. RSM is particularly advantageous because it reduces the number of experiments needed compared to conventional full factorial designs and enables the modeling of nonlinear behaviors through second-order polynomial equation (Eq. 1).

1 $$\begin{aligned} y &= \:{\beta\:}_{0} +\:\sum \limits _{i=1}^{k}{\beta\:}_{i}{X}_{i} + \sum \limits _{i=1}^{k}{\beta\:}_{ii}{X}_{i}^{2} +\sum \limits _{i,j=1}^{k.}{\beta\:}_{ij}{X}_{i}{X}_{j} \end{aligned}$$

In this study, RSM was employed to optimize the mechanical performance of epoxy-CNF composites. The independent variables selected were CNF content (A: 0.1, 0.55, and 1.0 wt%), mixing time (B: 10, 20, and 30 min), and curing temperature (C: 60, 80, and 100 °C). The responses under investigation were tensile strength and flexural strength. A three-factor, three-level BBD was employed to investigate the effects of these variables on the mechanical performance of the epoxy composites. The BBD was selected over other RSM approaches such as the Central Composite Design (CCD) due to its efficiency and suitability for the current experimental constraints. Specifically, BBD requires fewer runs compared to CCD for three variables, thereby reducing resource consumption and experimental workload. Furthermore, BBD avoids design points where all factors are simultaneously at their extreme levels, which was essential to prevent material degradation or processing challenges at high CNF loadings, long mixing durations, or elevated curing temperatures. This design ensured that all experimental conditions remained within practical and safe operational limits while allowing for the robust modelling of linear, interaction, and quadratic effects.

Design-Expert software was utilized to construct the experimental matrix, perform statistical analyses, and develop the regression models. ANOVA was conducted to evaluate the statistical significance of each model term and interaction effect. ANOVA is an essential tool in RSM, allowing for the identification of significant factors, interactions, and higher-order effects influencing the response variables. The significance of model terms was judged based on the F-values and p-values, where a high F-value and a p-value less than 0.05 indicated statistical significance. The normality of residuals was verified to ensure the validity of the ANOVA results, as assumptions regarding the distribution of residuals play a critical role in ensuring the reliability of statistical conclusions. Table 2 present the experimental design used for RSM analysis.

Based on the experimental results, second-order polynomial regression models were developed for both tensile and flexural strengths. The general form of the RSM-based quadratic model is presented in Eq. (2), where β₀ represents the intercept, β_i_ denotes the coefficients of the linear terms, β_i__i_ indicates the coefficients of the quadratic terms, and β_ij_ corresponds to the coefficients of the interaction terms.

2 $$\begin{aligned} y &= {\beta _0} + {\beta _1}A + {\beta _2}B + {\beta _3}C + {\beta _{11}}{A^2} + {\beta _{22}}{B^2} \\ & \quad + {\beta _{33}}{C^2} + {\beta _{12}}AB + {\beta _{13}}AC + + {\beta _{23}}BC \end{aligned}$$

**Table 2:** RSM experimental design (coded).

Ex no	CNF content	Mixing time	Curing temperature	Tensile strength (MPa)	Flexural strength (MPa)
1	1	0	1	61.78	74.36
2	1	0	− 1	40.48	45.48
3	− 1	0	− 1	26.19	31.5
4	− 1	1	0	42.92	47.33
5	0	0	0	49.47	60.51
6	0	0	0	50.1	60.97
7	− 1	− 1	0	17.3	24.67
8	1	− 1	0	45.61	57.54
9	0	0	0	50.3	60.26
10	0	− 1	− 1	29.01	31.77
11	0	0	0	48.58	59.58
12	0	0	0	49.66	59.2
13	0	− 1	1	36.84	50.4
14	0	1	− 1	40.89	44.17
15	0	1	1	59.26	65.93
16	1	1	0	52.08	58.97
17	− 1	0	1	33.06	42.46
18	0	0	0	49.81	60.3
19	0	0	0	49.97	58.83
20	0	0	0	51.14	60.75

## Results and discussion

### Experimental data analysis and interpretation

**Fig. 3:**
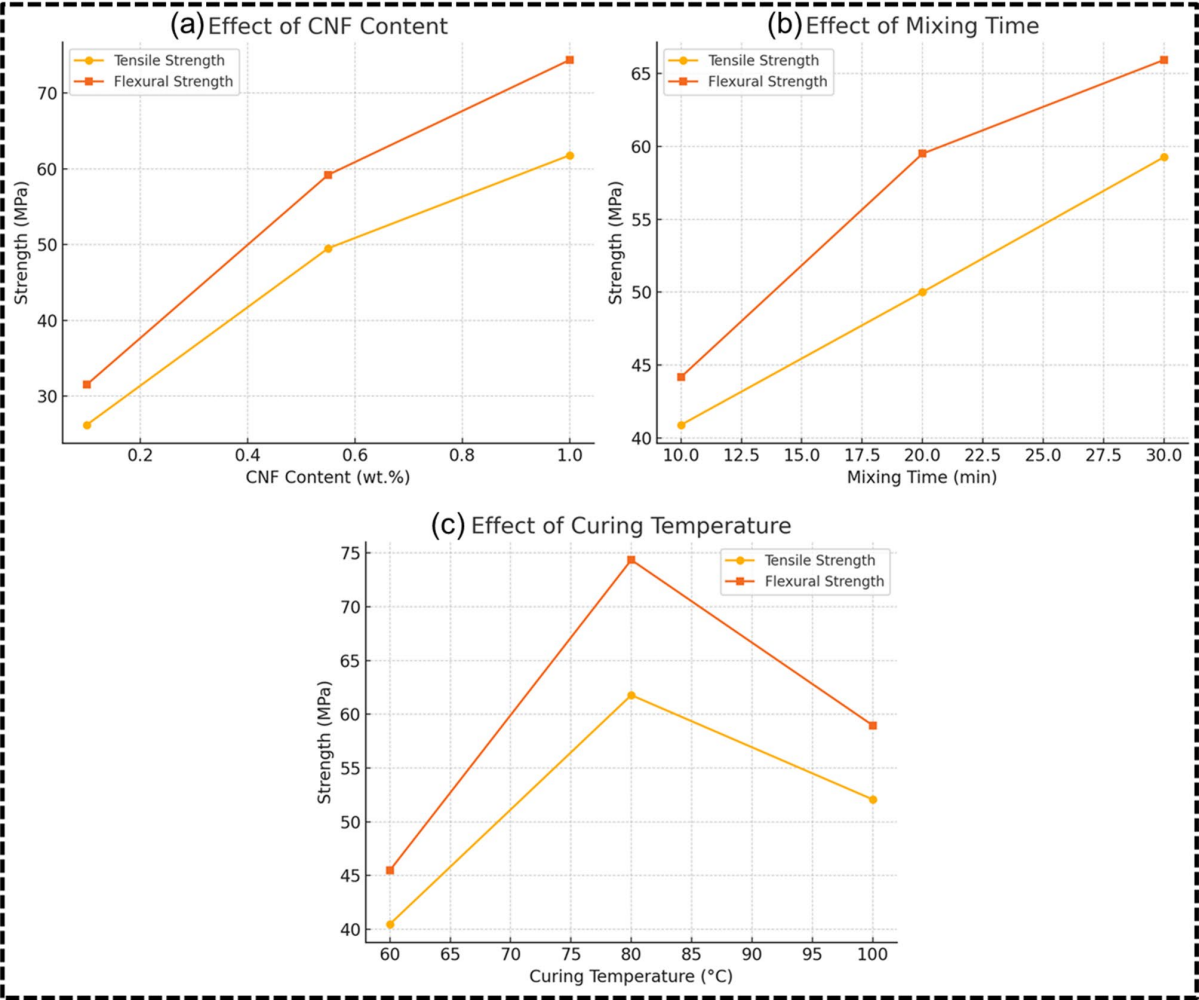
Effect of (**a**) CNF content, (**b**) mixing time, and (**c**) curing temperature on tensile and flexural strength of epoxy composites.

The mechanical performance of the CNF-reinforced epoxy composites was systematically evaluated by varying three critical processing parameters: CNF content (0.1 wt%, 0.55 wt%, and 1.0 wt%), mixing time (10, 20, and 30 min), and curing temperature (60 °C, 80 °C, and 100 °C). The results for tensile and flexural strengths presented in Fig. 3, demonstrate strong sensitivity to both individual and interactive effects of these parameters.

The incorporation of cellulose nanofibers had a profound influence on the mechanical properties of the composites. A general upward trend in both tensile and flexural strengths was observed with increasing CNF content from 0.1 wt% to 1.0 wt%. The highest tensile strength of 61.78 MPa and flexural strength of 74.36 MPa were recorded at 1.0 wt% CNF, 30-minute mixing time, and 80 °C curing temperature. This enhancement can be attributed to the superior mechanical characteristics of CNFs, high aspect ratio, nanoscale diameter, and excellent stiffness, which significantly improve stress transfer efficiency within the polymer matrix. However, the reinforcing effect of CNFs is highly dependent on their degree of dispersion and interfacial adhesion with the matrix. At low CNF loadings (0.1 wt%), insufficient fiber content limits the formation of an effective load-bearing network, leading to reduced mechanical response (e.g., 26.19 MPa tensile strength at 0.1 wt% CNF, 20 min mixing, and 60 °C curing). Moreover, excessive CNF content without proper dispersion mechanisms may lead to agglomeration, acting as stress concentrators and adversely affecting performance. Similar trends were reported by Saba et al.,^19^ where tensile strength increased up to 0.75% CNF loading but declined at 1% due to agglomeration. Their study highlighted that 0.75% CNF/epoxy composites exhibited optimal dispersion and interfacial adhesion. Beyond this, clustering of CNFs formed voids that reduced tensile performance.

#### Influence of mixing time

The influence of mixing time was directly related to the homogeneity of CNF dispersion within the epoxy matrix. An increase in mixing time from 10 to 30 min resulted in consistent improvement in both tensile and flexural properties. Extended mixing facilitates uniform nanofiber dispersion, reduces agglomerate size, and enhances wettability of CNFs by the epoxy resin. For example, at 0.55 wt% CNF and 80 °C curing, increasing mixing time from 10 to 30 min led to an increase in tensile strength from 40.89 MPa to 59.26 MPa, and flexural strength from 44.17 MPa to 65.93 MPa. These results suggest that sufficient shear energy input during mixing is essential to overcome fiber–fiber hydrogen bonding and to achieve nanoscale dispersion critical for reinforcing efficiency. In contrast, inadequate mixing (e.g., 10 min) often results in heterogeneous filler distribution, poor resin infiltration between fibers, and increased microvoid formation—leading to compromised mechanical integrity. These effects are especially evident when CNF content is high but the mixing time is short (e.g., 45.61 MPa tensile strength at 1.0 wt% CNF and 10 min mixing). A similar trend was observed by Pullicino et al.^20^, who demonstrated that shear mixing speed and time significantly affected agglomerate size and dispersion of graphene nanoplatelets in epoxy. At 3000 rpm for 2 h, they reported a 30% reduction in agglomerate size and a 14% increase in Young’s modulus. Their findings confirm that improved dispersion through optimal shear input enhances mechanical performance of nanoparticle-reinforced composites.

#### Influence of curing temperature

Curing temperature affects the crosslink density and thermal mobility of the epoxy chains, thereby influencing the matrix stiffness and interfacial bonding^21,22^. Across the experimental matrix, an optimal curing temperature of 80 °C emerged as the most effective condition for mechanical enhancement. For instance, composites cured at 80 °C consistently outperformed those cured at 60–100 °C when other factors were held constant. At 1.0 wt% CNF and 30 min mixing, curing at 80 °C resulted in 61.78 MPa tensile strength, while curing at 60 °C led to a drop to 40.48 MPa, and at 100 °C to 52.08 MPa. The superior performance at 80 °C can be attributed to controlled crosslinking kinetics that allow sufficient resin flow and fiber–matrix interaction prior to gelation. Curing at lower temperatures (60 °C) may result in incomplete polymerization and weaker network formation, while excessively high temperatures (100 °C) can cause thermal gradients, internal stresses, or even fiber degradation, leading to suboptimal mechanical performance. These observations are supported by the findings of Benedetti et al.^23^, which show that epoxy modulus and bond strength increase with curing temperature. Pull-out tests confirmed that elevated temperatures accelerate crosslinking, thereby improving stiffness and bonding performance in epoxy systems. Additionally, bond behaviour was found to stabilise after 24 h at 40 °C, highlighting the critical influence of curing temperature on mechanical maturity.

The experimental data also indicate the presence of synergistic effects when optimal levels of all three parameters are combined. The highest mechanical performance was consistently achieved under the combination of 1.0 wt% CNF content, 30 min of mixing, and 80 °C curing temperature, suggesting that the reinforcing potential of CNFs is fully realized only when adequate dispersion and sufficient interfacial bonding conditions are simultaneously met. This is further supported by the central point replicates (0.55 wt%, 20 min, 80 °C), which show consistent tensile and flexural strengths (approx. 49.5–51.1 MPa and 58.8–60.9 MPa, respectively), underscoring the reproducibility and stability of the process within the central region of the design space. Thus, it can be understood that the mechanical performance of CNF-reinforced epoxy composites is maximized through the concurrent optimization of CNF content, mixing time, and curing temperature. While CNF content acts as the primary reinforcement mechanism, its effectiveness is highly dependent on proper dispersion and network formation, governed by mixing and curing protocols. The observed trends emphasize that nano-reinforcement is a process-sensitive phenomenon, and achieving high-performance nanocomposites requires not only the right materials but also the correct processing strategies to unlock their full potential.

### Optimisation using RSM

#### Regression model analysis

The regression model for tensile strength exhibits a high coefficient of determination (R^2^ = 99.63%), indicating that more than 99% of the variability in tensile strength can be accurately explained by the model. The significant positive coefficients for CNF content (A) and mixing time (B) suggest that increasing these parameters enhances tensile strength, likely due to improved filler dispersion and interfacial adhesion between the matrix and CNF particles. Conversely, the negative coefficient for curing temperature (C) implies that excessive thermal exposure may reduce tensile strength, possibly due to polymer degradation, void formation, or thermal residual stresses. The significant interaction terms (AB, AC, BC) and quadratic terms (A^2^, B^2^, C^2^) reveal the non-linear behavior and synergistic effects among the input variables. Notably, the positive BC interaction indicates that combining longer mixing time with elevated curing temperatures may yield tensile strength improvements beyond the contributions of each factor individually. The flexural strength model also demonstrates excellent predictability, with an even higher R^2^ = 99.80%, validating the model’s robustness. The positive coefficients for CNF content (A) and curing temperature (C) indicate that both parameters strongly enhance flexural performance, likely by improving stiffness, crosslinking density, and interfacial load transfer. However, the negative coefficient for mixing time (B) suggests that excessive mixing may lead to CNF agglomeration or fiber breakage, ultimately reducing flexural strength. As in the TS model, the interaction and quadratic terms in the FS model emphasize complex multivariable influences. In particular, the positive AC interaction points to a favorable combined effect of CNF content and curing temperature on flexural performance. The developed regression equations are shown in Eqs. 3 and 4.

3 $$\begin{aligned} {\text{Tensile Strength }} & = {\text{ 49}}.{\text{87875 }} + {\text{ 1}}0.0{\text{6A }} + {\text{ 8}}.{\text{29875B }} \\ & \quad + {\text{ 6}}.{\text{79625C }}{-}{\text{ 4}}.{\text{7875AB }} + {\text{ 3}}.{\text{6}}0{\text{75AC }} + {\text{ 2}}.{\text{635BC}} \\ & \quad {\text{ }}{-}{\text{ 5}}.{\text{761875}}{{\text{A}}^{\text{2}}}{-}{\text{ 4}}.{\text{639375}}{{\text{B}}^{\text{2}}}{-}{\text{ 3}}.{\text{739375}}{{\text{C}}^{\text{2}}} \end{aligned}$$

4 $$\begin{aligned} {\text{Flexural Strength }} & = {\text{ 6}}0.0{\text{5 }} + {\text{ 11}}.{\text{29875A }} + {\text{ 6}}.{\text{5}}0{\text{25B }} \\ & \quad + {\text{ 1}}0.0{\text{2875C }}{-}{\text{ 5}}.{\text{3}}0{\text{75AB }} + {\text{ 4}}.{\text{48AC }} + {\text{ }}0.{\text{7825BC }} \\ & \quad -{\text{ 6}}.{\text{27}}{{\text{A}}^{\text{2}}}{-}{\text{ 6}}.{\text{6525}}{{\text{B}}^{\text{2}}}{-}{\text{ 5}}.{\text{33}}{{\text{C}}^{\text{2}}} \end{aligned}$$

### ANOVA results

To evaluate the statistical significance of the process parameters on the mechanical properties of the CNF-reinforced polymer composites, analysis of variance (ANOVA) was performed for both tensile strength and flexural strength responses. The results, summarized in Tables 3 and 4, confirm that the selected quadratic models are highly significant, offering reliable predictions within the experimental design space. For tensile strength, the model exhibited an F-value of 119.6 with a p-value < 0.0001, indicating a strong correlation with the experimental data. Among the input variables, CNF content emerged as the most influential parameter (F = 934.48), followed by mixing time (F = 635.91) and curing temperature (F = 426.49), with all effects showing statistical significance at *p* < 0.0001. These findings underscore the dominant role of filler concentration and process optimization in determining tensile behavior. Statistically significant interaction effects were also observed between CNF content and mixing time, as well as between CNF content and curing temperature (*p* < 0.01), revealing synergistic effects that further enhance tensile strength when parameters are tuned in combination. Additionally, the quadratic terms associated with CNF content, mixing time, and curing temperature demonstrated strong significance, indicating nonlinear behavior and the existence of optimal parameter levels.

Similarly, for flexural strength, the quadratic model was also found to be highly significant (F-value = 550.72, *p* < 0.0001). CNF content again exhibited the highest influence (F = 1646.71), followed by curing temperature (F = 1297.43) and mixing time (F = 545.40). The interaction terms between CNF content and both mixing time and curing temperature were significant, with F-values of 181.68 and 129.44, respectively, supporting the presence of combined effects that contribute positively to flexural performance. Although the interaction between mixing time and curing temperature was not statistically significant (*p* = 0.075), all three quadratic terms were highly significant, confirming curvature in the response surface and suggesting the existence of peak performance regions. Both models showed low residual errors and non-significant lack-of-fit values (tensile strength: *p* = 0.0994; flexural strength: *p* = 0.3749), indicating good model adequacy and predictive reliability. Moreover, the low pure error values affirm the reproducibility and consistency of the experimental results. These findings highlight that optimal mechanical performance can only be realized through precise, multivariable optimization of CNF content, mixing time, and curing temperature, which provides essential insights for the scalable development of high-performance, sustainable nanocomposites.

**Table 3:** ANOVA of TS.

Source	Sum of squares	df	Mean square	F value	*p*-value
Model	9653.4	9	1072.6	119.6	< 0.0001
A-CNF content	3560.1	1	3560.1	934.48	< 0.0001
B-Mixing time	2415.8	1	2415.8	635.91	< 0.0001
C-Curing temp	1620.3	1	1620.3	426.49	< 0.0001
AB	192.3	1	192.3	21.43	0.0004
AC	102.8	1	102.8	11.46	0.0048
BC	76.5	1	76.5	8.53	0.0121
A^2^	285.9	1	285.9	31.87	0.0002
B^2^	198.2	1	198.2	22.09	0.0007
C^2^	139.5	1	139.5	15.56	0.0025
Residual	89.7	10	8.97		
Lack of fit	84.2	5	16.84		0.0994
Pure error	5.5	5	1.1		

**Table 4:** ANOVA of FS.

Source	Sum of squares	df	Mean square	F-value	*p*-value
Model	3074.0	9	341.56	550.72	< 0.0001 significant
A-CNF content	1021.29	1	1021.29	1646.71	< 0.0001
B-Mixing time	338.26	1	338.26	545.4	< 0.0001
C-Curing temperature	804.61	1	804.61	1297.43	< 0.0001
AB	112.68	1	112.68	181.68	< 0.0001
AC	80.28	1	80.28	129.44	< 0.0001
BC	2.45	1	2.45	3.95	0.075
A^2^	179.72	1	179.72	289.77	< 0.0001
B^2^	291.76	1	291.76	326.2	< 0.0001
C^2^	129.87	1	129.87	209.44	< 0.0001
Residual	6.2	10	0.62		
Lack of fit	2.12	3	0.7052	1.21	0.3749
Pure error	4.09	7	0.5838		
Cor total	3080.2	19			

**Fig. 4:**
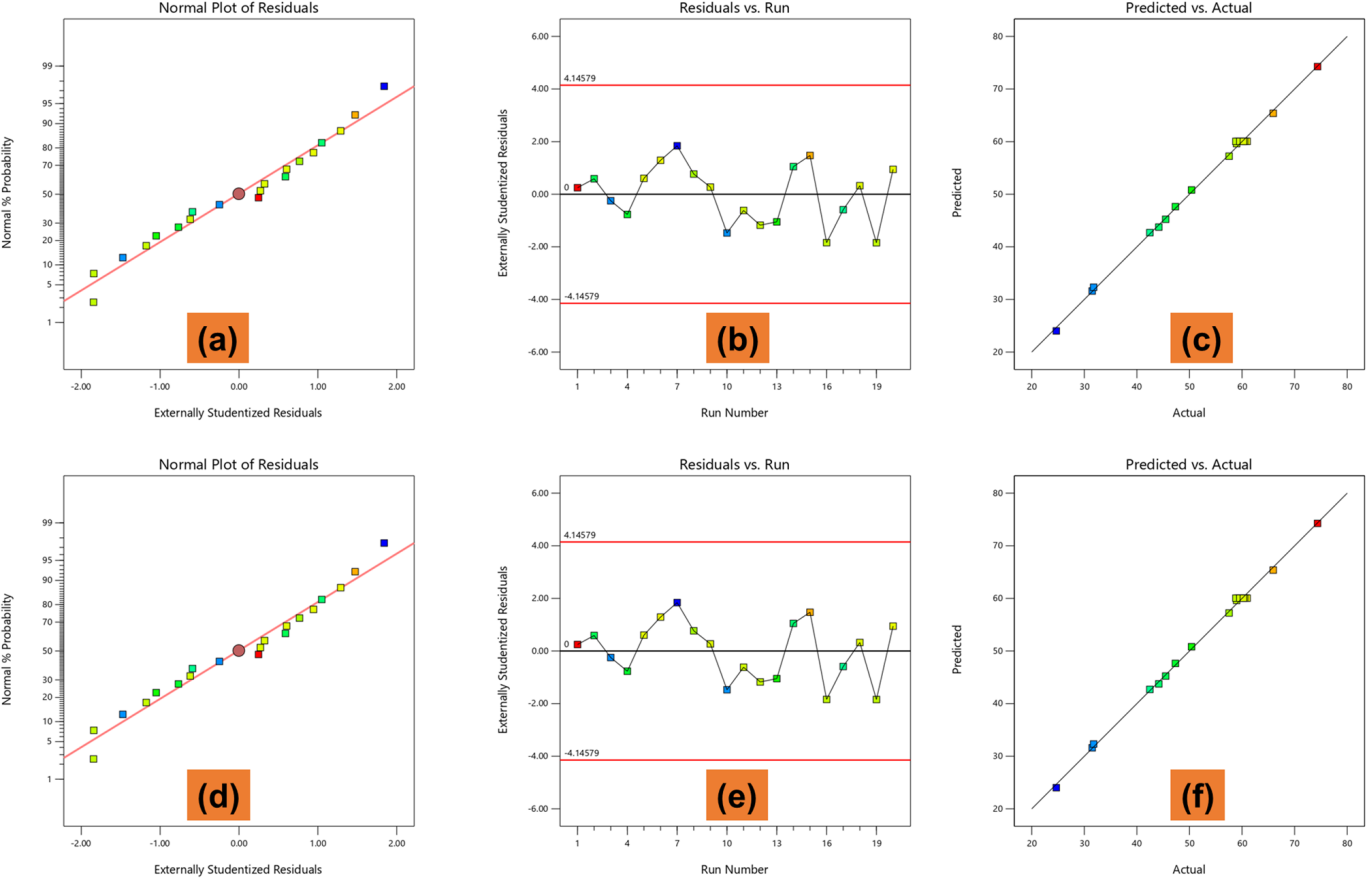
Diagnostic plots: (**a**–**c**) Tensile strength; (**d**–**f**) Flexural strength, showing residual normality, run order independence, and predicted vs. actual agreement.

To assess the reliability and adequacy of the developed quadratic regression models for tensile strength and flexural strength, diagnostic plots were examined, as shown in Fig. 4. For tensile strength, the normal probability plot of residuals (Fig. 4a) shows that the externally standardized residuals follow a straight line, indicating normal distribution of errors and supporting the model’s validity. The residuals versus run plot (Fig. 4b) displays a random scatter around zero with no observable trend, confirming the absence of systematic bias or heteroscedasticity. All residuals fall within the control limits, suggesting no significant outliers. Furthermore, the predicted versus actual plot (Fig. 4c) reveals a tight clustering of data points along the diagonal reference line, affirming excellent model predictability with minimal deviation. Similar observations were made for the flexural strength model. The normal probability plot (Fig. 4d) again confirms the normality of residuals, while the residuals versus run plot (Fig. 4e) shows a random, pattern-free distribution of residuals around the baseline, further validating the independence and constant variance assumptions. In the predicted versus actual plot (Fig. 4f), the data points lie close to the ideal fit line, indicating high agreement between observed and predicted flexural strength values. These diagnostic results strongly support the statistical robustness of both regression models and align with the high R^2^ values (99.63% for tensile strength and 99.80% for flexural strength), adjusted R^2^ values (99.30% and 99.62%), and predicted R^2^ values (99.43% and 98.73%) reported earlier. Collectively, these findings confirm the suitability of the developed models for accurate prediction and optimization of mechanical properties in CNF-reinforced polymer composites.

### Interaction effects on tensile and flexural strength

**Fig. 5:**
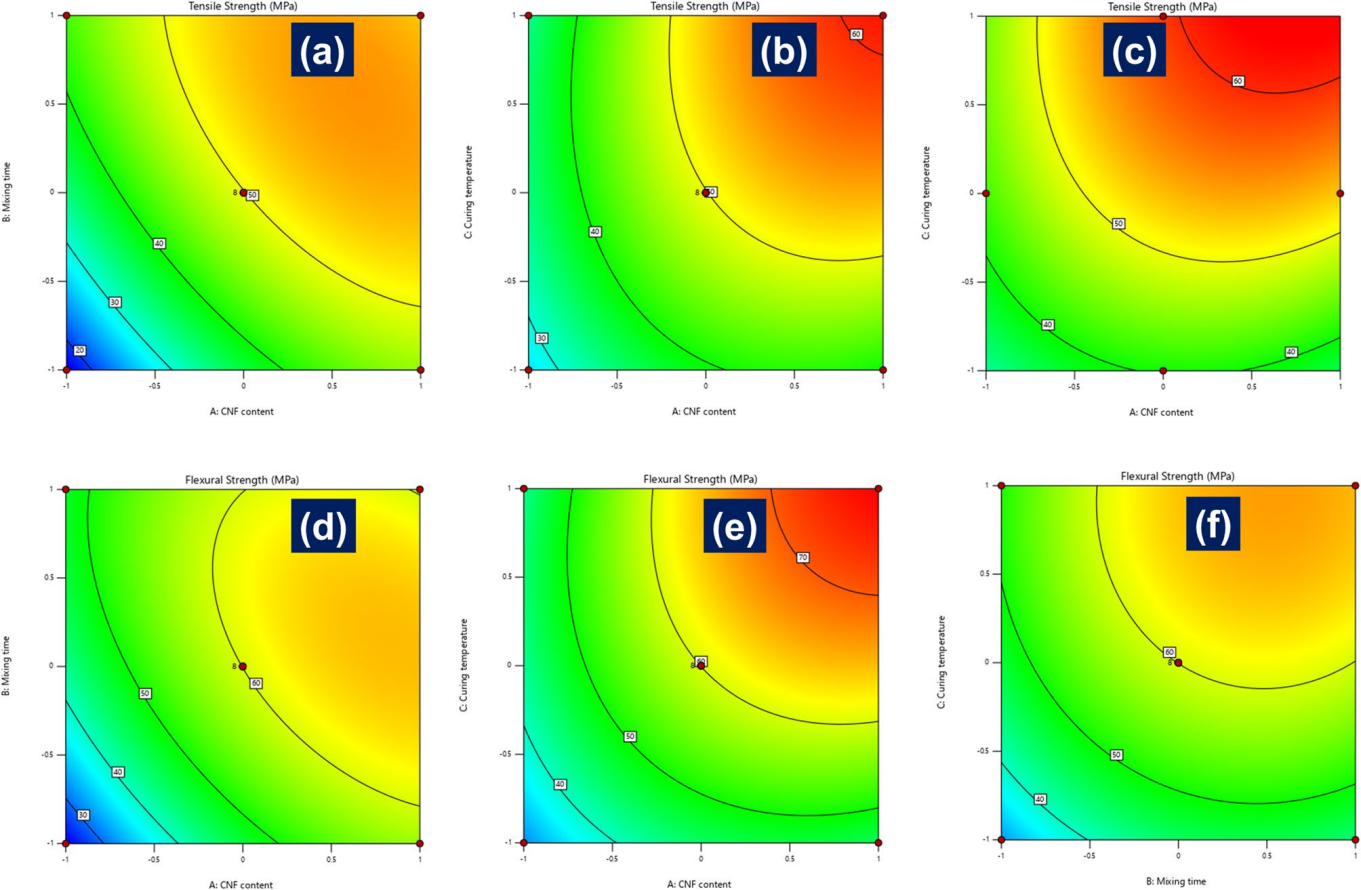
Interaction effects on tensile and flexural strength.

The interaction effects of the process parameters, CNF content, mixing time, and curing temperature, on the mechanical responses were further analyzed using two-dimensional contour plots for tensile strength (Fig. 5a–c) and flexural strength (Fig. 5d–f). These plots reinforce the quadratic behavior of the fitted models and provide a visual representation of the synergistic relationships among the input variables. In the case of tensile strength, the contour plot of CNF content versus mixing time (Fig. 5a) exhibits a clear synergistic effect, with strength values increasing significantly in regions where both parameters are high. This enhancement is attributed to improved nanofiber dispersion at longer mixing durations and the reinforcing effect of CNFs, which facilitate superior stress transfer within the matrix. Similarly, the contour plot of CNF content versus curing temperature (Fig. 5b) indicates tensile strength peaks at high CNF loadings combined with elevated curing temperatures, suggesting improved interfacial bonding and increased crosslink density. However, the curvature in the contours suggests that excessively high curing temperatures may lead to thermal degradation or internal stress development, which could marginally reduce performance. The interaction between mixing time and curing temperature (Fig. 5c) also yields a moderate yet notable enhancement in tensile strength, indicating that sufficient mixing combined with optimized curing is critical for effective CNF distribution and matrix hardening.

A similar pattern is observed in the flexural strength response. The contour plot of CNF content and mixing time (Fig. 5d) demonstrates that flexural strength improves significantly with higher levels of both parameters, consistent with the hypothesis that enhanced dispersion and interfacial adhesion contribute to improved bending resistance. The interaction between CNF content and curing temperature (Fig. 5e) shows a pronounced increase in flexural strength, likely due to combined effects of nanofiber reinforcement and thermally induced matrix crosslinking, which enhance stiffness and rigidity. Although the mixing time/curing temperature interaction (Fig. 5f) is statistically less significant, the general trend still indicates an upward trajectory in flexural strength with increasing curing temperature and moderate mixing durations. Overall, the contour plot analyses confirm that mechanical properties are influenced not only by the individual contributions of CNF content, mixing time, and curing temperature but also by their coupled interactions, which must be jointly optimized to achieve superior composite performance.

**Fig. 6:**
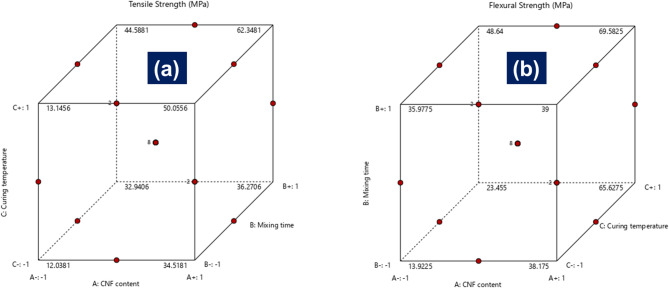
Cube plots were for (**a**) tensile strength and (**b**) flexural strength.

To further investigate the interactive influence of the process parameters on mechanical performance, cube plots were generated for both tensile and flexural strength responses, as shown in Fig. 6. These plots illustrate the predicted outcomes at all factorial combinations of CNF content, mixing time, and curing temperature, offering a concise yet insightful representation of three-way interactions. In the case of tensile strength, the lowest predicted value (12.04 MPa) occurred at the vertex corresponding to the lowest levels of all three parameters, namely, 0.1 wt% CNF content, 10 min mixing time, and 60 °C curing temperature. This configuration likely results in poor fiber dispersion, weak interfacial adhesion, and incomplete matrix crosslinking, collectively degrading mechanical performance. Conversely, the maximum tensile strength (62.35 MPa) was predicted at the combination of 1.0 wt% CNF, 30 min mixing, and 100 °C curing temperature, indicating a strong synergistic effect when optimal reinforcement loading, adequate mixing energy, and appropriate thermal curing are applied concurrently.

Transitions along the cube edges further reveal the sensitivity of tensile performance to individual parameters when the other two are fixed at high levels. For instance, increasing CNF content from 0.1 wt% to 1.0 wt% while maintaining long mixing time and high curing temperature led to an improvement of nearly 18 MPa, affirming the dominant reinforcing effect of well-dispersed CNFs. The center point, corresponding to 0.55 wt% CNF, 20 min mixing, and 80 °C curing, yielded an intermediate tensile strength of 50.06 MPa, validating the model’s curvature and supporting the use of a second-order regression approach.

A similar trend was observed in flexural strength predictions. The lowest value (13.92 MPa) was again observed under the least favorable conditions, minimal CNF loading, short mixing time, and low curing temperature, while the highest value (69.58 MPa) was achieved at the optimal combination of 1.0 wt% CNF, 30 min mixing, and 100 °C curing. This significant enhancement highlights the critical importance of simultaneously optimizing all three parameters. Notably, flexural strength was especially responsive to increases in curing temperature when CNF content was high, suggesting a key role for thermal crosslinking in matrix rigidity. Moreover, the cube plot edges illustrate that nonlinear interaction effects exist between the parameters, with the greatest improvements seen when multiple variables are elevated in unison. The center point for flexural strength, predicted at 39 MPa, represents a balanced performance outcome and reinforces the validity of the experimental design.

### Numerical optimization, desirability analysis, and validation

**Fig. 7:**
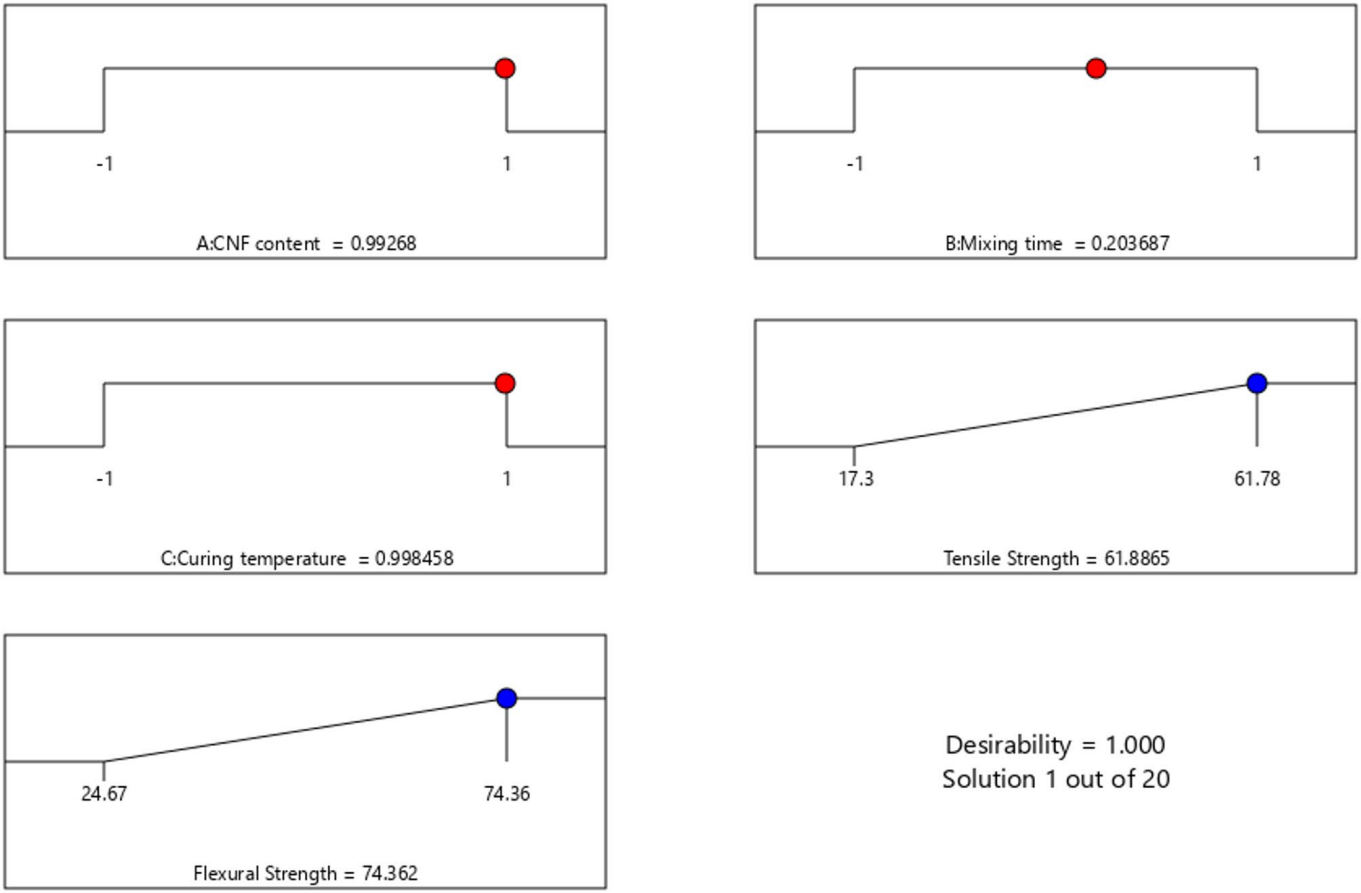
Desirability ramp plot for multi-objective optimization.

The optimal combination of process parameters for maximizing the mechanical performance of CNF-reinforced epoxy composites was determined using multi-objective optimization via the desirability function approach and results is presented in Table 5. The optimization aimed to simultaneously maximize tensile strength and flexural strength. As illustrated in Fig. 7, the highest-ranking solution (Solution 1 out of 100) achieved a composite desirability score of 1.000, indicating full satisfaction of the optimization criteria. The optimal parameter combination included a CNF content of ≈ 1.5 wt%, a mixing time of ≈ 22 min, and a curing temperature of ≈ 80 °C. These values correspond to the coded values of 0.993, 0.204 and 0.998, respectively. Based on the developed regression models, the predicted tensile and flexural strengths at these conditions were 61.88 MPa and 74.36 MPa, respectively.

**Table 5:** Optimum values.

Factor	Value
CNF content	1.498 ≈ 1.5 wt%
Mixing time	22.13 ≈ 22 min
Curing temperature	79.96 ≈ 80 °C

To validate the optimization outcome, a confirmatory experiment was conducted under the predicted optimal settings. The experimentally obtained tensile strength was 58.62 MPa, while the flexural strength was 76.41 MPa. These values were in reasonable agreement with the model predictions, 61.88 MPa for tensile strength and 74.36 MPa for flexural strength, showing deviations of 5.27% and 2.76%, respectively (Table 6). This validation confirms the predictive accuracy and robustness of the RSM-based models. It further demonstrates that moderate CNF loading, extended mixing duration, and controlled curing at lower temperatures are effective strategies for enhancing composite performance. Additionally, the optimization framework presented here supports the practical scalability of CNF-based nanocomposite fabrication and highlights the value of statistical modelling in developing sustainable, high-performance materials.

**Table 6:** Comparison of predicted and experimental values at optimal conditions.

Response	Predicted value (MPa)	Experimental value (MPa)	% Deviation
Tensile strength	61.88	58.62	5.27%
Flexural strength	74.36	76.41	2.76%

## Conclusion

This study demonstrates the successful extraction and incorporation of cellulose nanofibers (CNFs) derived from areca nut husk as reinforcing agents in epoxy composites, offering a sustainable and value-added approach to agricultural waste utilisation. The mechanical performance of the CNF-reinforced epoxy was found to be highly sensitive to the processing parameters,specifically, CNF content, dispersion time, and curing temperature. Optimisation using a statistically robust Response Surface Methodology (RSM) model identified optimal conditions at approximately 1.5 wt% CNF content, 22 min of mixing time, and a curing temperature of 80 °C. Under these conditions, the composite exhibited tensile and flexural strengths of 61.88 MPa and 74.36 MPa, respectively. Experimental validation confirmed the model’s reliability, with deviations of 5.27% (tensile) and 2.76% (flexural), thereby affirming the predictive accuracy of the RSM approach. ANOVA results highlighted the statistical significance of both linear and interaction effects, with low residual errors and a non-significant lack-of-fit (*p* > 0.09), further supporting the model’s adequacy. Given the favourable mechanical properties and the renewable nature of the reinforcement, the developed composites are promising candidates for applications in automotive interior components, electronic casings, and non-load-bearing structural parts. Beyond demonstrating the feasibility of areca nut husk valorisation, this work establishes a scalable route for tailoring the performance of bio-based nanocomposites. Future studies may explore hybridisation with alternative nanofillers or chemical surface modifications to enhance interfacial interactions and expand the utility of such composites in demanding functional environments.

## Data Availability

The datasets generated and/or analysed during the current study are available from the corresponding author on reasonable request.
